# Buckwheat Flower Volatiles Attract *Peristenus spretus* and Enhance Its Field-Level Parasitism of *Apolygus lucorum*

**DOI:** 10.3390/plants12081658

**Published:** 2023-04-15

**Authors:** Shike Xia, Tao Zhang, Livy Williams, Yizhong Yang, Yanhui Lu

**Affiliations:** 1 State Key Laboratory for Biology of Plant Diseases and Insect Pests, Institute of Plant Protection, Chinese Academy of Agricultural Sciences, Beijing 100193, China; 2 College of Plant Protection, Yangzhou University, Yangzhou 225007, China; 3 Key Laboratory of IPM on Crops in Northern Region of North China, Ministry of Agriculture and Rural Affairs, Institute of Plant Protection, Hebei Academy of Agricultural and Forestry Sciences, Integrated Pest Management Center of Hebei Province, Baoding 071000, China; 4 USDA-ARS U.S. Vegetable Laboratory, Charleston, SC 29414, USA; 5 Western Agricultural Research Center, Chinese Academy of Agricultural Sciences, Changji 831100, China

**Keywords:** Miridae, Braconidae, parasitoids, volatiles, behavioral response, electrophysiological response, field trapping

## Abstract

Volatile compounds play indispensable roles in the interactions among host plants, herbivores and natural enemies. Previous studies showed that the addition of buckwheat strips in cotton fields could attract *Peristenus spretus*, the dominant parasitoid of *Apolygus lucorum*, and enhance its parasitic activity. Through the combined analysis of Y-tube olfactometer, solid-phase microextraction (SPME), gas chromatography-mass spectrometer (GC-MS) and electroantennography (EAG), we found that male and female *P. spretus* responded to compounds present in buckwheat flowers. The five major components of buckwheat flowers, cis-3-hexenyl acetate (Z3HA), 4-methylanisole, 4-oxoisophorone, p-methylphenol and 2-ethylhexyl salicylate, all had a significant attraction to *P. spretus* adults and led to positive electroantennogram responses, especially for 10 mg/mL 4-oxoisophorone, indicating the components played a key role in the selection behavior of *P. spretus* to buckwheat flowers. Additionally, field trials showed that the five volatiles could significantly increase the parasitism by *P. spretus*. Our study screened the key active components of buckwheat flower volatiles that have an attractive effect on *P. spretus*, revealing its behavioral selection mechanism and emphasizing the important role of plant volatiles on host selection and parasitism of parasitic wasps, providing a theoretical basis for the development of attractants for *P. spretus* and the reduction of pesticides in the field to promote conservation biological control (CBC) of *A. lucorum*.

## 1. Introduction

Conservation biological control (CBC) refers to protecting natural enemies or improving their control ability by changing the field environment or pesticide application methods [1]. Compared with classical or augmentative biological control, CBC pays more attention to the conservation and protection of natural enemy insect populations [2], which is an effective way to achieve sustainable pest control and maintain ecological balance. Utilizing the volatile organic compounds (VOCs) to attract natural enemies, increasing the number of attracted natural enemies or establishing a “Push-Pull” system [3,4] are major research focuses of CBC.

VOCs are volatile secondary substances continuously released by plants during their growth and development processes [5], playing an irreplaceable role in maintaining the ecological balance in tritrophic interactions among host plants, herbivorous insects and natural enemies. VOCs provide herbivores with cues for long-distance host identification and orientation, acquisition of nutritional resources and mating sites, as well as guidance on oviposition and mating behaviors [6]. In addition, VOCs can act as information substances for natural enemy searching behavior [6,7,8,9]. In corn fields, nerolidol, cis-jasmone and trans-β-farnesene were effective in attracting natural enemies [10]. Three volatiles from the pagoda tree *Sophora japonica* L., α-pinene, linalool and hexanal at the concentrations of 10^−4^~10^−6^ g/mL, 10^−4^ g/mL and 10^−5^ g/mL, respectively, were significantly attractive to the ladybug, *Harmonia axyridis* (Pallas), an important natural enemy of insect pests such as scale insects and aphids [11]. The volatiles bornylene, 6-methyl-5-hepten-2-one and (+)-6-methyl-5-hepten-2-ol induced by wheat aphid were the most important chemical signaling substances for the parasitoid *Aphidius avenae* Haliday, as well as predatory *Coccinella septempunctata* L., *Propylaea japonica* (Thunberg), *Chrysoperla sinica* (Tjeder) and *Chrysopa pallens* (Rambur) to search for their hosts [12,13].

The mirid bug, *Apolygus lucorum* (Hemiptera: Miridae), is one of the most important pests of cotton, cereals, vegetables and fruit crops in China, especially in the Yangtze River Basin and the Yellow River Basin [14]. *A. lucorum* adults and nymphs can damage host plants through piercing and sucking feeding activities, and their wide environmental adaptability, high population growth rate and strong dispersal ability can lead to a loss in cotton yield of 20–30% in some years [15]. *A. lucorum*, especially males, had a strong electrophysiological response to butyrate and green leaf volatiles (GLVs) [16]. Previous studies showed that fragrant volatiles emitted by flowers could mediate the preference of *A. lucorum* for flowering host plants [17]. Moreover, temporal shifts in plant volatile emission may modulate host plant foraging of *A. lucorum* and appear to guide its colonization of different host plants [18]. When applied to fields, flower-emitted VOCs acted as attractants for several mirids, including *Adelphocoris suturalis*, *Ad. lineolatus* and *Ad. fasciaticollis* [19].

*Peristenus spretus* Chen *et* van Achterberg (Hymenoptera: Braconidae) is a key solitary koinobiont endoparasitoid native to China, with a distribution range of 23–39° N [20,21]. When *P. spretus* was released in field cages at the rate of one female parasitoid per 50 *A. lucorum* nymphs, the parasitism rate reached 77.8% [22]. The foraging behavior of natural enemies can be regulated by olfactory cues, such as the odor produced by flowers and vegetative plant parts [2,23]. *P. spretus* females use volatiles released by plants infested by *A. lucorum* nymphs to locate host plants with potential hosts, and the behavioral attraction of *P. spretus* to flowering host plants is similar to that of adult *A. lucorum* [24].

Buckwheat, *Fagopyrum esculentum* Moench, is a valuable nectary source for parasitoids and also a common grain crop in China with a long flowering period and well-developed nectaries [25]. Buckwheat is widely used in CBC due to its value to improve the performance of parasitic wasps [23,26]. The introduction of flowering plants in or around cultivated fields has been used to enhance the control of pests by natural enemies [27]. For example, adding 2 m-wide buckwheat strips in cotton fields attracted *P. spretus* and enhanced their parasitic activity [28]. We hypothesize that buckwheat flower volatiles are a key factor in the attraction of buckwheat to parasitic wasps and may have a positive effect on their function in the field. Further, knowledge of the bioactive volatiles produced by buckwheat might permit use of synthetic analogs to attract *P. spretus* to crop fields without sacrificing part of the field for buckwheat production.

In this study, we used a Y-tube olfactometer, solid-phase microextraction (SPME), gas chromatography-mass spectrometer (GC-MS) and electroantennography (EAG) to assess the behavioral and physiological responses of *P. spretus* adults to volatiles produced by buckwheat flowers under laboratory and field conditions.

## 2. Results

### 2.1. Behavioral Responses to Buckwheat Plants

**Figure 1:**
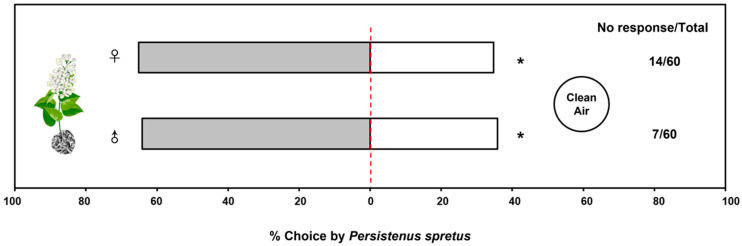
Behavioral response of *Peristenus spretus* adults to buckwheat plants in a Y-tube olfactometer. Data in the horizontal bar chart are the percentage of individuals that responded in each treatment out of the total number of individuals that responded, and the numbers next to the chart indicate the number of individuals who did not respond to either treatment out of the total individuals in the trial. 0.01 < *p* < 0.05 (*) means significantly difference.

The behavioral responses of adult wasps to volatiles from buckwheat flowers in a Y-tube olfactometer were assessed and analyzed, indicating that volatile odors from buckwheat flowers have a significant trapping effect on both male (64.15%) and female (65.22%) *P. spretus* (female: *χ*^2^ = 4.26, *df* = 1, *p* = 0.04; male: *χ*^2^ = 4.25, *df* = 1, *p* = 0.04) compared with clean air (Figure 1).

### 2.2. Analysis of Buckwheat Volatiles

**Figure 2:**
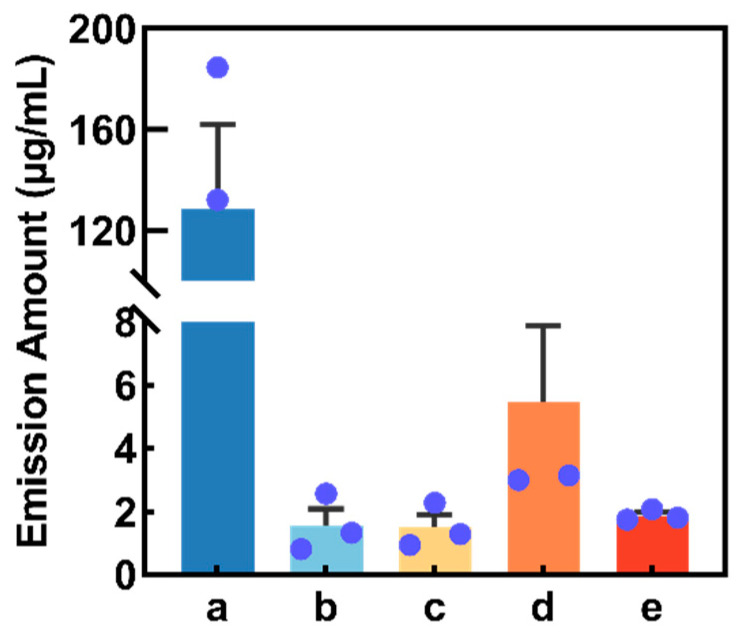
Concentration of volatile compounds collected from buckwheat flowers (4 h collection period). The blue dot in the figure represents the original data. (a) cis-3-Hexenyl acetate. (b) 4-Methylanisole. (c) 4-Oxoisophorone. (d) p-Methylphenol. (e) 2-Ethylhexyl salicylate.

After collecting buckwheat flower volatiles through SPME technology, the major substances in buckwheat flower volatiles were screened and identified by GC-MS, and five compounds were obtained, while compounds often associated with air (e.g., toluene and benzene) or laboratory equipment (e.g., siloxanes or phthalates) were not included in our list of putative plant volatiles [29]. According to the sequence of GC retention time (RT), they were (1) cis-3-hexenyl acetate (Z3HA, RT = 10.07 min), (2) 4-methylanisole (RT = 11.12 min), (3) 4-oxoisophorone (RT = 16.30 min), (4) p-methylphenol (RT = 24.93 min) and (5) 2-ethylhexyl salicylate (RT = 29.16 min). Additionally, the compound Z3HA had the greatest concentration (128.31 ± 33.60 mg/mL), followed by p-methylphenol (5.48 ± 2.41 mg/mL), 2-ethylhexyl salicylate (1.87 ± 0.11 mg/mL), 4-methylanisole (1.56 ± 0.52 mg/mL) and 4-oxoisophorone (1.49 ± 0.4 mg/mL) (Figure 2).

### 2.3. EAG Responses to Single Compounds

**Figure 3:**
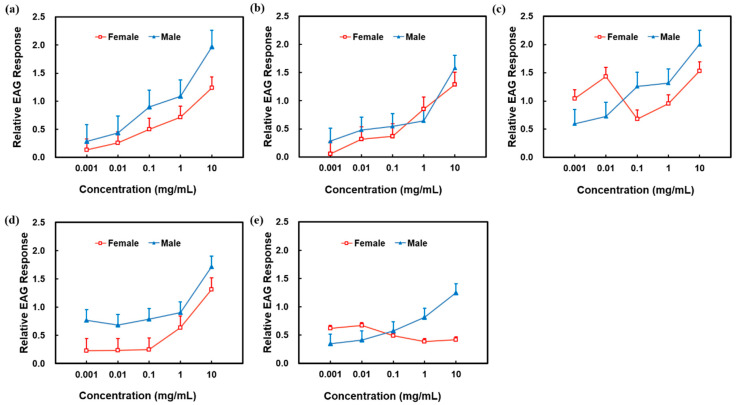
EAG response of *Peristenus spretus* antennae to buckwheat flowers. EAG relative value = (EAG amplitude of the test stimulus − mean EAG amplitude of the control)/(mean EAG amplitude of the reference compound − mean EAG amplitude of the control). (**a**) cis-3-Hexenyl acetate. (**b**) 4-Methylanisole. (**c**) 4-Oxoisophorone. (**d**) p-Methylphenol. (**e**) 2-Ethylhexyl salicylate.

The EAG response relative value of male *P. spretus* increased with increasing concentrations for all compounds identified by GC-MS and reached the maximum value at 10 mg/mL for 4-oxoisophorone. Equally, the EAG response relative value for female *P. spretus* increased with the increase of the concentrations of Z3HA, 4-methylanisole and p-methylphenol and was the largest at 10 mg/mL 4-oxoisophorone. However, the EAG amplitude response of females decreased at 0.1 mg/mL 4-oxoisophorone, as well as 0.1 and 1 mg/mL 2-ethylhexyl salicylate (Figure 3).

### 2.4. Behavioral Responses to Principal Compounds

**Figure 4:**
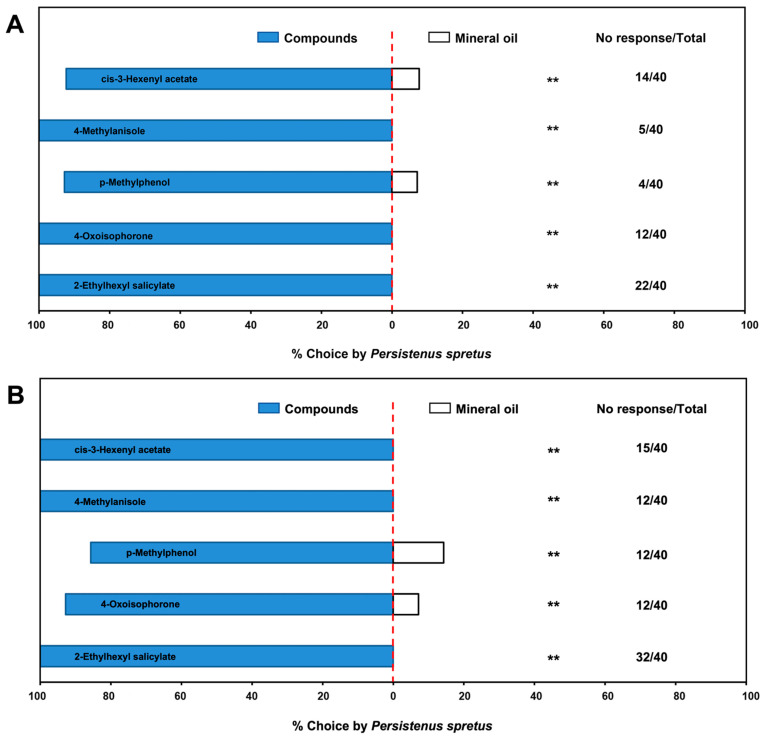
Behavioral response of *Peristenus spretus* males (**A**) and females (**B**) to main components of buckwheat flower volatiles in a Y-tube olfactometer. Data in the horizontal bar chart are the percentage of individuals that responded in each treatment out of the total number of individuals that responded, and the numbers next to the chart indicate the number of individuals who did not respond to either treatment out of the total individuals in the trial. *p* < 0.01 (**) means highly significantly different.

In a Y-tube olfactometer, the above-mentioned five electrophysiological volatiles all had significant attraction to male (Z3HA: *χ*^2^ = 18.62, *df* = 1, *p* < 0.01; 4-methylanisole: *χ*^2^ = 35.00, *df* = 1, *p* < 0.01; p-methylphenol: *χ*^2^ = 36.00, *df* = 1, *p* < 0.01; 4-oxoisophorone: *χ*^2^ = 20.57, *df* = 1, *p* < 0.01; 2-ethylhexyl salicylate: *χ*^2^ = 18.00, *df* = 1, *p* < 0.01) and female (Z3HA: *χ*^2^ = 25.00, *df* = 1, *p* < 0.01; 4-methylanisole: *χ*^2^ = 28.00, *df* = 1, *p* < 0.01; p-methylphenol: *χ*^2^ = 20.57, *df* = 1, *p* < 0.01; 4-oxoisophorone: *χ*^2^ = 14.29, *df* = 1, *p* < 0.01; 2-ethylhexyl salicylate: *χ*^2^ = 8.00, *df* = 1, *p* < 0.01) parasitoids. Thus, all five compounds played key roles in the preference for buckwheat flowers by *P. spretus* (Figure 4).

### 2.5. Parasitism in the Field

**Figure 5:**
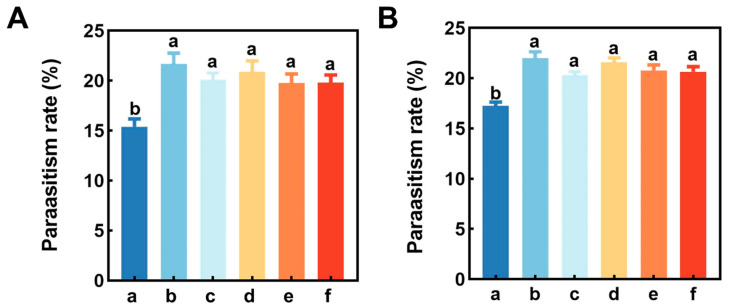
Parasitism rate of *Apolygus lucorum* nymphs by *Peristenus spretus* wasps in barrel traps baited with 10 mg/mL concentration of buckwheat major volatile compounds in cotton field (**A**) and vineyard (**B**) test areas. We have added the meaning of the lower case letters above the bar chart. (a) Mineral oil, (b) cis-3-Hexenyl acetate, (c) 4-Methylanisole, (d) 4-Oxoisophorone, (e) p-Methylphenol, (f) 2-Ethylhexyl salicylate. Letters above the bars indicate significant differences.

Field trials showed that the five main components of buckwheat flower volatiles significantly enhanced parasitism of *A. lucorum* nymphs by *P. spretus* in both cotton fields and vineyards (cotton field: *F* = 5.92, *df* = 5, 71, *p* < 0.001; vineyard: *F* = 12.46, *df* = 5, 71, *p* < 0.001). In cotton fields and vineyards, the five volatiles of buckwheat flowers increased parasitism by 28.25~40.78% and 18.05~27.86%, rezspectively, with the most significant potentiation of parasitism by Z3HA (Figure 5).

## 3. Discussion

Plant volatiles play an important role in the trophic relationship, not only providing beneficial and herbivorous insects with clues related to food orientation, habitat location and oviposition sites but also helping predatory and parasitic natural enemies locate their hosts, thus enabling plants to engage in indirect defense or enhance their direct resistance to herbivores [30,31]. In addition, plants and herbivores often determine the physiological and behavioral responses of parasitic wasps. In this study, we found the flowers of the nectar source plant, buckwheat, were significantly attractive to *P. spretus* adults. Further, we identified five floral volatiles (Z3HA, 4-methylanisole, 4-oxoisophorone, p-methylphenol and 2-ethylhexyl salicylate) from buckwheat flowers, all of which had electrophysiological and behavioral activities on both male and female *P. spretus* adults, indicating that these compounds played a key role in the attraction of *P. spretus* to buckwheat flowers. Our results suggest that integration of buckwheat strips into crop fields may enhance CBC of *A. lucorum* by *P. spretus*.

The main components of buckwheat flower volatiles were Z3HA and p-methylphenol which were confirmed to be attractive to *P. spretus* males and females through the behavioral determination of electrophysiological volatiles. P-Methylphenol is often used as bait for traps due to its enticing effect on pests and parasitic wasps [32]. Z3HA is one of the dominant GLVs emitted by wounded plants [33], as well as the main component of some floral volatiles, especially in the HS-SPME of flowers, such as *Jasminum sambac* [34], *Rubus idaeus* [35], *Lonicera japonica* (Thunberg) [36] and living rose [37]. In addition, the attraction effect of Z3HA on parasitic wasps has also been demonstrated in other parasitoids. *Campoletis chlorideae* Uchida is an endophytic wasp of *Helicoverpa armigera* (Hübner). Z3HA not only had an attractive effect on the parasitoid, but a specific dose of Z3HA significantly increased parasitism of *H. armigera* by *C. chlorideae* [38]. Thus, use of synthetic analogs of plant volatiles which are attractive to natural enemies has potential to improve pest control without sacrificing cropland, which is of great significance in CBC. Further field studies are necessary to evaluate the efficacy of synthetic attractants based on buckwheat volatiles for *A. lucorum* control by *P. spretus*.

Insect olfaction varies with compound type and concentration [39] and the species and sex of organism [40]. In general, EAG amplitude increased with ascending concentrations for the five compounds within a certain range. The EAG response relative value of male *P. spretus* was the maximum when the concentrations of Z3HA, 4-methylanisole, 4-oxoisophorone, p-methylphenol and 2-ethylhexyl salicylate were 10 mg/mL, and that of females reached the maximum when the concentrations of Z3HA, 4-methylanisole, 4-oxoisophorone and p-methylphenol were 10 mg/mL while 2-ethylhexyl salicylate was at 0.01 mg/mL. In summary, the EAG response of *P. spretus* could be affected by the type and concentration of volatile compounds, which is an important guideline for selecting suitable compounds and concentrations to make attractants for biological control of *A. lucorum* in the field.

Insects mainly recognize external chemical signals through olfaction; their olfactory receptors are mainly distributed on antennae where chemical communication among the tritrophic levels is mainly accomplished [41]. This process also requires the involvement of multiple proteins, such as odorant-binding proteins (OBPs), chemosensory proteins (CSPs), odorant receptors (ORs) and ionotropic receptors (IRs) [42]. Competitive binding experiments found that Z3HA had high binding affinity to *Agos*OBP8, which can participate in olfactory and taste recognition at the same time, playing a physiological role in regulating behavior of *Aphis gossypii* [43]. In addition, SlituOR12, which could affect the detection of host location and oviposition site of moth females, with expressed oocytes having extremely high sensitivity to Z3HA [41]. It could be inferred that Z3HA plays an important role in the host recognition process of insects and affects their searching behavior.

Previous laboratory studies revealed that feeding on buckwheat flowers could significantly increase the daily parasitism rate of *P. spretus* by 39.8% compared with water [25]. In addition, Li et al. [28] evaluated the parasitism rate of *A. lucorum* in field plots of 13 plant species and found that the parasitism rate among buckwheat flowers was the highest, i.e., 2.9× that of cotton. Additionally, we here demonstrated that volatile compounds of buckwheat flowers could also significantly increase the parasitism rate of *P. spretus* under field conditions. Our results provide insight on the role of buckwheat floral VOCs in *P. spretus* behavior and enable future studies toward the development of a CBC program for *A. lucorum*.

## 4. Materials and Methods

### 4.1. Biological and Chemical Materials

#### 4.1.1. Insects

Mirid bugs. Adults and nymphs of *A. lucorum* were obtained from Langfang Experimental Station, Chinese Academy of Agricultural Sciences (CAAS), Hebei Province, China (116°36′7″ E, 39°30′31″ N). These insects were continuously reared in 20 × 10 × 6 cm transparent plastic boxes at 25 ± 1 °C, 60 ± 5% RH and 14:10 h (L:D) photoperiod. Adults of *A. lucorum* were provided with fresh green bean pods (*Phaseolus vulgaris* L.), rinsed with sodium hypochlorite (NaClO) solution (0.5%), as both food and an oviposition substrate, whereas nymphs were reared on kernels of organic corn (*Zea mays* L.) [15,44].

Parasitoids. *P. spretus* adults were obtained from a laboratory colony at Langfang Experimental Station, CAAS, where this parasitoid had been continuously reared for >1 year in plexiglass rearing cages (30 × 30 × 25 cm) at 25 ± 1 °C, 60 ± 5% RH and 14:10 h (L:D) photoperiod, using 3rd to 5th instar nymphs of *A. lucorum* as hosts while corn kernels continued to be offered for consumption [20].

#### 4.1.2. Plants

Buckwheat seeds were sown in the growth medium mixed with peat soil: vermiculite: medium loam in the ratio of 6: 1:1 (by volume) in a greenhouse at Langfang Experimental Station, CAAS, under the following conditions: 26 ± 1 °C, 60 ± 10% RH and 14:10 h (L:D) photoperiod. Cotton (CCRI49) seeds were obtained from the Institute of Cotton Research of CAAS and sown in a field at Langfang Experimental Station of CAAS in May, and no fertilizers, pesticides or herbicides were used on the plants.

### 4.2. Behavioral Bioassays with Buckwheat Flowers

**Figure 6:**
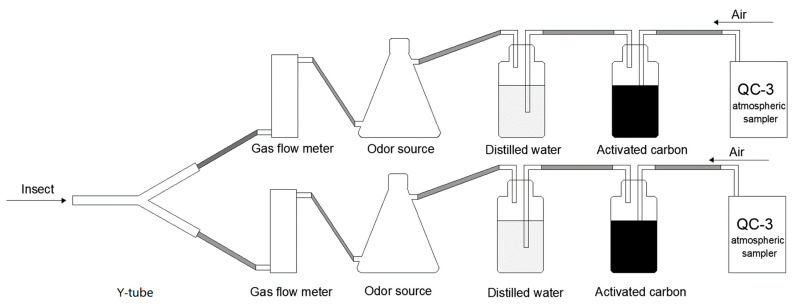
Schematic of Y-tube olfactometer.

Referring to Yu et al. [14] and Xiu et al. [45], a Y-type olfactory device (inner diameter: 2 cm; length of main and selector arms: 15 cm; angle between selector arms: 60°) was used to evaluate the behavioral responses of *P. spretus* adults to buckwheat flowers. A QC-3 atmospheric sampler (Beijing Municipal Institute of Labor Protection, Beijing, China) was used as the airflow power source to connect activated Z3HArcoal, a distilled water humidification device, a gas flow control meter, a glass odor source vessel and the Y-tube test arena with Teflon tubes and to seal the connections with parafilm (Figure 6).

Two pots of full-bloom buckwheat flowers were selected to conduct our olfactometer bioassays at 25 ± 1 °C from 900 h to 1700 h. Before the test, the buckwheat plants were rinsed with ultra-pure water; the soil part was wrapped tightly with tin foil; and care was taken to avoid plant damage. An intact buckwheat plant was then placed into one odor source vessel, and the other odor source was an empty air control.

One active, unmated and healthy 2-day-old *P. spretus* adult (*n* = 60 males and 60 females) was introduced to the initial test chamber. When parasitoid wasps crossed 1/3rd of either test arm within 5 min and remained there for more than 10 s, it was considered to be a selection; if wasps did not respond as such they were considered to be unresponsive and discarded. After testing five parasitic wasps, the two arms of the Y-tube were reversed, and after testing 10 wasps a clean Y-tube was used. At the end of the test, the Y-tube, odor source vessel and Teflon tubes were washed with 95% ethanol, soaked and rinsed with distilled water and air-dried at room temperature.

### 4.3. Collection and Analysis of Buckwheat Volatiles

Buckwheat flower volatiles were collected by SPME. Prior to the start of the experiment, the SPME device was activated in the GC-MS injection port for 30 min (230 °C). Latex gloves were worn to pick buckwheat flowers from plants, which accounted for about 1/3rd of the transparent glass sample bottle (10 mL). The sample bottle was sealed and placed horizontally on the lab bench. SPME collections were then made for 4 h with a fiber needle coated with quartz (INNOTEG, Beijing, China), and a total of three biological replicates were set up.

GC-MS (GC: Agilent 7890A, equipped with a DB-WAX chromatographic column [30 m × 0.25 mm × 0.25 µm]; MS: Agilent 5975C) was used to identify and analyze the major substances of buckwheat flowers. GC and MS working conditions were similar parameters as those in previous studies [45]. The injector temperature for GC analysis was 230 °C; the oven temperature was kept at 50 °C for 1 min, then raised by 5 °C/min to 180 °C for 2 min, then increased by 10 °C/min to 230 °C and held for 2 min. Helium was the carrier gas, at an average flow rate of 1 mL/min. The ion source temperature was 230 °C. The volatile samples were first identified automatically by NIST 14, and then their retention times and external standard method were, respectively, used for compound identification and qualitative analyses. Some 1 mL five standard compounds at different concentrations (0.001, 0.002, 0.05, 0.1 and 0.2 mg/mL) which were dissolved in hexane, respectively, were used to establish an external standard curve, whose *y*-axis was the peak areas of mass chromatograms, and *x*-axis was the substance content (mg/L). The emission amount of buckwheat flower volatiles can be obtained by substituting the peak area of the corresponding compound.

### 4.4. EAG Recordings

Electroantennographic tests were performed with an EAG detector system (Syntech Ltd., Hilversum, The Netherlands) with similar parameters as those in Liu [46] and Zhang et al. [47], including the control recording of electrodes, amplifier and signal electronic processing, signal display and recording system, and stimulus application system.

Active 2-day-old *P. spretus* adults (*n* = 3 males and 3 females for each treatment) with intact antennae were selected to assess their antennal responses to the volatiles of buckwheat flowers. One antenna was cut off along at the base with a scalpel, and the tip of the antenna was also removed before the antenna was placed between the reference electrode and recording electrode. charcoal filtered, humidified air (0.4 L/min) was blown over the antenna for a few minutes to allow the baseline to stabilize. Then, the electrodes were inserted into the EAG probe for signal acquisition, amplification and conversion analysis. The order of the test solutions was: control (paraffin oil), five standard compounds at different concentrations (0.001, 0.01, 0.1, 1 and 10 µg/µL) which were dissolved in paraffin oil and the standard reference compound (10 µg/µL cis-3-hexen-1-ol) [47]. Ten µL of each test solution was placed on a rectangular filter paper strip (0.5 cm × 4 cm) and placed in a glass Pasteur pipette for presentation to the antenna. The duration of each stimulation was 0.5 s, and the interval between two stimuli was 30 s during which time clean humidified air was blown over the antenna. Each antenna was stimulated seven times.

### 4.5. Behavioral Bioassays with Synthetic Compounds

The Y-tube olfactometer was used to evaluate behavioral responses of 2-day-old active *P. spretus* adults (*n* = 40 males and 40 females for each treatment) to major components of buckwheat flower volatiles. One side of the test tube was mineral oil as control, while the other side was respectively placed with (1) 10 mg/mL Z3HA, (2) 10 mg/mL 4-methylanisole, (3) 10 mg/mL 4-oxoisophorone, (4) 10 mg/mL p-methylphenol and (5) 10 mg/mL 2-ethylhexyl salicylate. Ten μL of each test solution was pipetted onto the filter paper strip and put into the odor source bottle, following protocols as above.

### 4.6. Field Trials

Field parasitism rate trials were conducted in August 2022 in cotton fields (0.13 ha) and vineyards (0.11 ha), respectively, located at Langfang Experimental Station, CAAS. During the experimental period, no pesticides were applied. Prior to each trial (replicate), bamboo poles with barrel traps (Insect collector: upper diam: 16.5 cm; lower diam: 13.0 cm; height: 12.5 cm; Pherobio Technology Co., Ltd., Beijing, China) were randomly placed in each test plot at 10 m distances, with each trap 20 cm from the top of the plants. A dilution of 200 µL of five volatiles (Z3HA, 4-methylanisole, 4-oxoisophorone, p-methylphenol, 2-ethylhexyl salicylate) at a concentration of 10 mg/mL was separately placed in a rubber septum (length: 1.84 cm; upper diameter: 0.89 cm; lower diameter: 0.46 cm) in each trap, and mineral oil was used as the control. Each barrel trap was filled with 100 2nd–3rd instar *A. lucorum* nymphs and four beans as food source. Then, a total of 50 pairs of 2-day-old *P. spretus* adults were released at the center and four corners of each plot. Two days later, *A. lucorum* nymphs were transferred indoors and fed with corn for 6 d until they were dissected to assess *P. spretus* parasitism. Five volatiles and one control were placed in each of the six traps in the field to test effects of different volatile compounds on parasitism by the wasps, and 12 trials (replicates) were conducted in cotton fields and vineyards respectively.

### 4.7. Statistical Analysis

Chi-square tests were used to analyze the Y-tube olfactometer data to detect differences between the pairs of treatments. *χ*^2^ and P values were calculated, and non-responsive adults were excluded from the analysis. The emission amounts, relative EAG response of each volatile and field parasitism rates were compared using one-way ANOVA, followed by Duncan’s new multiple range tests. Chi-square tests and one-way ANOVAs were conducted using SPSS 25.0.

## 5. Conclusions

From the perspective of chemical ecology, our study found that buckwheat flowers and their five active volatile components (Z3HA, 4-methylanisole, 4-oxoisophorone, p-methylphenol and 2-ethylhexyl salicylate) had a significant attraction on *P. spretus* adults through the combined analysis of Y-type insect olfactometer, SPME, GC-MS and EAG. In addition, this study clarified the EAG response of buckwheat flowers on *P. spretus* and the key role of buckwheat flowers in the selection behavior of *P. spretus*; the antennal potential response of males and females was especially the strongest under 10 mg/mL of 4-Oxoisophorone. Moreover, field experiments revealed the positive effect of five volatiles on the parasitism of *P. spretus*, which could increase the parasitism rate by ca. 40%. This study illustrated the selective behavior of *P. spretus* on buckwheat flowers and its chemical recognition mechanism for buckwheat flower volatiles, which provided a theoretical basis for the development of *P. spretus* attractants and CBC of *A. lucorum*. Further work is necessary to evaluate the potential benefits of buckwheat volatiles to feral *P. spretus* and to determine the relative attractiveness of volatiles from buckwheat flowers versus synthetic volatiles on *P. spretus* attraction and parasitism.

## Data Availability

Not applicable.
